# Transcriptional regulator-mediated activation of adaptation genes triggers CRISPR *de novo* spacer acquisition

**DOI:** 10.1093/nar/gku1383

**Published:** 2015-01-07

**Authors:** Tao Liu, Yingjun Li, Xiaodi Wang, Qing Ye, Huan Li, Yunxiang Liang, Qunxin She, Nan Peng

**Affiliations:** 1 State Key Laboratory of Agricultural Microbiology, College of Life Science and Technology, Huazhong Agricultural University, Wuhan 430070, P. R. China; 2 Hubei Collaborative Innovation Center for Industrial Fermentation, Wuhan 430070, P. R. China; 3 Archaeal Centre, Department of Biology, University of Copenhagen, Ole Maaløes Vej 5, DK-2200 Copenhagen N, Denmark

## Abstract

Acquisition of *de novo* spacer sequences confers CRISPR-Cas with a memory to defend against invading genetic elements. However, the mechanism of regulation of CRISPR spacer acquisition remains unknown. Here we examine the transcriptional regulation of the conserved spacer acquisition genes in Type I-A of *Sulfolobus islandicus* REY15A. Csa3a, a MarR-like transcription factor encoded by the gene located adjacent to *csa1, cas1, cas2* and *cas4* cluster, but on the reverse strand, was demonstrated to specifically bind to the *csa1* and *cas1* promoters with the imperfect palindromic sequence. Importantly, it was demonstrated that the transcription level of *csa1, cas1, cas2* and *cas4* was significantly enhanced in a *csa3a-*overexpression strain and, moreover, the Csa1 and Cas1 protein levels were increased in this strain. Furthermore, we demonstrated the hyperactive uptake of unique spacers within both CRISPR loci in the presence of the *csa3a* overexpression vector. The spacer acquisition process is dependent on the CCN PAM sequence and protospacer selection is random and non-directional. These results suggested a regulation mechanism of CRISPR spacer acquisition where a single transcriptional regulator senses the presence of an invading element and then activates spacer acquisition gene expression which leads to *de novo* spacer uptake from the invading element.

## INTRODUCTION

Clustered regularly interspaced short palindromic repeats (CRISPR) and their associated genes (*cas*) constitute the CRISPR-Cas immune system, which takes up DNA from invasive genetic elements to serve as a memory source that provides adaptive defense against mobile genetic elements (1,2). The CRISPR-Cas system is widespread and is found in about 90% of archaeal and 40% of bacterial genomes (3). The CRISPR loci consists of identical repeats (typically 20–50 bp in length) interspaced by variable spacer sequences of similar size (4). Repeats are usually conserved in sequence within a given locus and often contain partially palindromic sequences (5). Spacers, in contrast, are highly diverse in sequence and originate from mobile genetic elements (4,6–8), which led to the hypothesis that CRISPRs provide immunity against invading genetic elements (9). An AT-rich leader sequence located upstream of the first repeat promotes transcription of the CRISPR locus (10,11). The *cas* genes encode a diverse family of proteins carrying nuclease, helicase and potential polymerase activities (9,12), and based on the diversity of Cas proteins, CRISPR-Cas systems have been clustered into three basic types: Type I, Type II and Type III, which are further divided into at least 11 subtypes (13). Although most *cas* genes are highly divergent and are only associated with certain CRISPR loci, *cas1* and *cas2* are notably conserved in sequence across the three major types of CRISPR systems (2).

The mechanism by which CRISPR-Cas provides immunity against mobile genetic elements can be disected in three steps: (i) incorporation of an invading nucleic acid sequence into the CRISPR locus; (ii) CRISPR-RNA (crRNA) transcription and maturation; and (iii) target interference by crRNA and a protein complex of Cas proteins, which is guided by crRNA base-pairing (14,15). Although the processes of crRNA biogenesis and interference are well characterized, spacer acquisition mechanisms remain poorly understood. The first successful demonstration of spacer acquisition under laboratory conditions was in the *Streptococcus thermophilus* Type II-A system. In this study, addition of new repeat-spacer units were found and the new spacer perfectly matched the sequence of the challenging phage (1). Subsequent studies identified more spacer acquisition events in different CRISPR-Cas types, including *Escherichia coli* Type I-E (16–18), *Pseudomonas aeruginosa* Type I-F (19), *Sulfolobus solfataricus* Type I-A (20,21), *Haloarcula hispanica* Type I-B (22,23) and *Pectobacterium atrosepticum* Type I-F (24). A spacer acquisition assay demonstrated that Cas1 and Cas2 are the proteins required for new spacer adaptation at the host CRISPR locus (17,22,25,26). A protospacer-adjacent motif (PAM) of efficient protospacer-mediated spacer acquisition was identified in *E. coli* and *H. hispanica* (18,23). Moreover, other DNA motifs, such as CRISPR leader sequences and priming spacers, play important roles in spacer acquisition (17,23,25,27–29). Recently it was found that the presence of defective phages favors spacer acquisition in *S. thermophilus* (30). It is worthy to know Heler *et al*. have reviewed the current knowledge of the CRIPSR spacer acquisition mechanism (31). Although transcriptional regulation of CRISPR array by Cbp1 in *Sulfolobus* (32) and *cas* genes in bacteria by a cyclic AMP receptor protein, heat-stable nucleoid-structuring protein (H-NS) and LeuO, a LysR-type transcription regulator (10,33–36) have been reported, the transcriptional regulation of the most conserved genes *cas1* and *cas2*, which are involved in new spacer acquisition, needs much to be learn.

The archaeon *Sulfolobus islandicus* REY15A encodes one adaptation module and three CRISPR interference modules, one of Type I-A and two of Type III-B (37). The CRISPR-Cas system in this genus has recently been investigated with respect to the dispensability of its *cas* genes for proper CRISPR function (38), dynamic properties of CRISPR loci after plasmid challenge (39) and the mechanism of transcription-dependent DNA interference (37). In this study, we present *in vivo* and *in vitro* evidence for Csa3a activating the transcription of *cas* genes that are involved in new spacer acquisition (a*cas* genes), thereby triggering spacer acquisition within both CRISPR loci in *S. islandicus*.

## MATERIALS AND METHODS

### Strains and growth conditions

*Sulfolobus islandicus* E233S (*ΔpyrEF ΔlacS*) was cultured in SCVy medium at 78°C (40). Electroporation was used to transform *S. islandicus* E233S, and transformants were selected on 2-layer phytal gel plates as described previously (40). *E. coli* DH5α and Rosetta cells were used for DNA cloning and recombinant protein production. All *E. coli* strains were cultured at 37°C in Luria-Bertani medium and ampicillin or kanamycin was added to reach a 100 μg/ml or 30 μg/ml final concentration as required.

### General DNA manipulation methods

Restriction and DNA modification enzymes were purchased from New England Biolabs or Fermentas. Plasmid DNA was extracted from *E. coli* or *Sulfolobus* cells using an AxyPrep Plasmid Miniprep Kit (Wujiang, China). Polymerase chain reaction (PCR) products were purified by using an Axygen PCR Clean-up Kit, and DNA bands fractionated from the agarose gel were extracted by using an Axygen DNA extraction kit. Total DNA was prepared from *Sulfolobus* by using an Axygen Genomic DNA Miniprep Kit. The oligonucleotides were synthesized by Invitrogen (Shanghai, China) and were also used for DNA sequencing.

### Protein expression and purification

The *csa3a, csa1, cas1, cas2* and *cas4* genes were amplified from *S. islandicus* REY15A genomic DNA using the primers listed in Supplementary Table S1, and cloned into *E. coli* expression vector pET30a or *Sulfolobus* expression vector pSeSD (41). After amplification in *E. coli* DH5α, the expression plasmids were transformed into *E. coli* Rosetta cells or *S. islandicus* E233S. For protein expression in *E. coli*, the recombinant proteins were induced at 20°C for 8 h by addition of 0.8 mM isopropyl β-d-1-thiogalactopyranoside (IPTG) at the final concentration. After induction, cells were harvested and sonicated. The soluble proteins in the supernantants collected by centrifugation at 12 000 ×*g* were purified by using a nickel matrix. After dialysis against 10 mM Tris–HCl buffer (pH 8.0), the proteins were either used immediately or stored at −80°C. The protein concentration was determined by using the MicroBCA kit (Pierce; Rockford, IL, USA) according to the manufacturer's instructions.

### Electrophoretic mobility shift assay

Electrophoretic mobility shift assay (EMSA) probes were generated by PCR or annealing using the oligonucleotides with one of the primer pair 5′-end biotin-labeled (Supplementary Table S1). Then the products were purified from 6% native polyacrylamide gel electrophoresis (PAGE). The EMSA binding reactions (20 μl) containing 10 pmol of biotin-labeled probes and different concentrations of Csa3a, as described in the figure legends, were incubated for 20 min at 40°C in the binding buffer (20 mM Tris–HCl, pH8.0, 50 mM KCl, 5% glycerol, 1 mM ethylenediaminetetraacetic acid, 1 mM dithiothreitol, 5 ng/μl poly(dI-dC)). For specific competition, increasing amounts of unlabeled specific probes were added to the reaction mixture. After the reaction, samples were loaded onto a 6% native PAGE gel buffered with 1× TBE solution. DNA–protein complexes were separated at 100 V for 90 min and then transferred to a polyvinylidenefluoride membrane (Bio-Rad, Hercules, CA, USA) using the Semi-Dry Electrophoretic Transfer Cell system (Bio-Rad). Bands were visualized by chemiluminescent detection using the clarity Western ECL substrate (Bio-Rad, Hercules, CA, USA) and the MF-Chemibis 3.2 imaging device (DNR; Jerusalem, Israel).

### DNase I footprinting assay

A DNase I footprinting assay was performed as described previously (42) with slight modifications. The *csa1* and *cas1* promoter sequences were amplified from genomic DNA using primer sets *csa1*F-*csa1*R (–205 to –3, related to translational start codon) and *cas1*F-*cas1*R (–143 to +97), and subcloned into pMD18T (Takara, Dalian, China). The promoter DNA sequences were PCR-amplified from the pMD18T plasmids carrying *csa1* and *cas1* promoter sequences using primers with either 5′-end 6-HEX-labeled M13F (for the coding strand) or 5′-end FAM-labeled M13R (for the non-coding strand, Supplementary Table S1), and purified by 6% PAGE. In a 60 μl reaction system, 100 ng of a labeled DNA fragment was bound to 100 μg Csa3a (bovine serum albumin was used instead of Csa3a in the control experiment) in the buffer containing 10 mM Tris–HCl, pH7.4, 10 mM MgCl_2_, 1 mM CaCl_2_, 0.4 mM dithiothreitol, 100 mM KCl and 5% glycerol, and incubated for 20 min at 40°C. After binding, 0.03 U of RNAse-free DNase I (Roche, Basel, Switzerland) was added and allowed to react for 5 min at 30°C. The reaction was stopped and precipitated with ethanol. Samples were analyzed in a 3730 DNA Analyzer (Applied Biosystems, Foster City, CA, USA) and the electropherograms were aligned with GeneMapper v3.5 (Applied Biosystems).

### Chromatin immunoprecipitation assay

A chromatin immunoprecipitation (ChIP) assay was performed as described previously (43) using rabbit polyclonal antibody against Csa3a. The DNA from ChIP samples was extracted and resolved in 50 μl TE buffer. Input samples were treated following the same procedure without addition of antiserum and Protein A + G agarose beads (Beyotime, Beijing, China). Recovered DNA was PCR-amplified using primers specific for the *csa1, cas1* and *lrs14* (SiRe_0993) promoters (Supplementary Table S1). The recovered DNA was further analyzed by quantitative PCR. Each quantification reaction contained 1 μl recovered DNA, 1 μl (10 μM) gene-specific forward and reverse primers (the same primers as used above) and 10 μl SsoFast EvaGreen Supermix (Bio-Rad, Hercules, CA, USA) in a final volume of 20 μl. The reactions were run on a LightCycler 480 (Roche, Basel, Switzerland). The *lrs14* promoter sequence quantification was used as negative control. The qPCR conditions were as follows: 30 s of enzyme activation at 95°C, followed by denaturing at 95°C for 5 s and annealing/extension at 60°C for 20 s. The samples were cooled to 50°C and then heated to 99°C, and the melting curves were determined. Data analysis was performed using the software Roche LightCycler 480 HTC1. The ChIP assay were done in triplicate.

### Construction of a *S. islandicuscsa3a*-deletion mutant

The gene deletion method recently developed for *S. islandicus* REY15A (44) was employed to knockout the *csa3a* gene in this study. The left sequence arm (L-arm), right sequence arm (R-arm) and target gene arm (G-arm) were amplified from *S. islandicus* REY15A using the primer set shown in Supplementary Table S1. The marker cassette sequence carrying the *pyrEF* and *lacS* genes was amplified from the *Sulfolobus–E. coli* shuttle vector pHZ2lacS(40) using the primers M-F-BamHI and M-R-SalI. All the fragments were subsequently cloned onto vector pUC19, resulting in the *csa3a* gene-deletion plasmid pDel*csa3a* (Supplementary Figure S1). The selection of *csa3a-*deletion mutants using this plasmid was carried out as described previously (44).

### Total RNA preparation

Total RNA was isolated from exponentially growing *Sulfolobus* cultures in SCVy medium or ACVy medium for induction of *csa3a* gene under control of *araS* promoter (OD_600_ = 0.2) as described previously (45). Genomic DNA in the total RNA sample was removed using DNase I (Roche, Basel, Switzerland).

### Reverse transcription (RT)-PCR and real-time RT-PCR (RT-qPCR) assay

**Figure 1.**
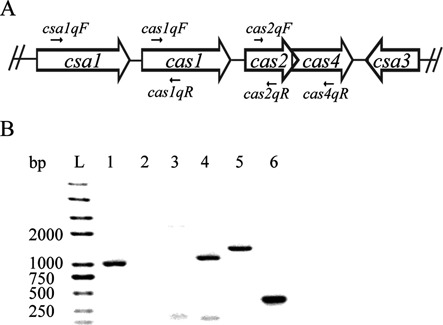
Determination of the co-transcription of the *csa1, cas1, cas2* and *cas4* genes. (**A**) Organization of *csa1* (852 bp), *cas1* (873 bp), *cas2* (270 bp) and *cas4* (528 bp) genes is depicted schematically. The locations of primers are indicated that were used for RT-PCR to determine co-transcription. The intergenic regions between *csa1* and *cas1* is 42 bp and between *cas1* and *cas2* is 110 bp; *cas2* and *cas4* are overlapped. (**B**) Fragments obtained in RT-PCR experiments carried out with the primers *csa1*qF and *cas1*qR (lane 1, product 1016 bp), *csa1*qF and *cas2*qR (lane 2), *csa1*qF and *cas4*qR (lane 3), *cas1*qF and *cas2*qR (lane 4, product 1151 bp), *cas1*qF and *cas4*qR (lane 5, product 1418 bp), and *cas2*qF and *cas4*qR (lane 6, product 376 bp). A DNA ladder (Trans 2K plus II) was run in lane L as a size marker.

To determine the co-transcription of the *csa1, cas1, cas2* and *cas4* genes, three gene-specific reverse primers (*cas1*qR, *cas2*qR and *cas4*qR; Supplementary Table S1, Figure 1) were used to generate the first-strand cDNA from the total RNA sample. Three forward primers (*csa1*qF, *cas1*qF and *cas2*qF; Supplementary Table S1, Figure 1) in combination with the three reverse primers listed above were used to amplify the first-stand cDNAs (Figure 1). The PCR products were separated on a 1% agarose gel. For real-time reverse transcription-PCR (RT-qPCR), first-strand cDNA was synthesized using M-MuLV reverse transcriptase (Promega, Madison, WI, USA) and the specific reverse primers (*csa1*qR, *cas1*qR, *cas2*qR and *cas4*qR; Supplementary Table S1). Each real-time quantification reaction was carried out as described above for the ChIP-qPCR assay using the first-strand cDNAs as template and each forward primer (*csa1*qF, *cas1*qF, *cas2*qF, *cas4*qF or *csa3a*qF) in combination with the reverse primers listed above along with *csa3a*qR. The transcripts of the *albA* gene were used as the control (45) and the cycle threshold (Ct) values of the control transcript *albA* were used to normalize the Ct values of the *csa1, cas1, cas2, cas4* and *csa3a* transcripts.

### Western blotting

*Sulfolobus* transformants were cultured in ACVy medium to induce expression of *csa3a* gene under control of *araS* promoter. When OD_600_ reached 0.2, cells were harvested and sonicated. Crude protein samples were separated by 12% sodium dodecyl sulphate (SDS)-PAGE, and then transferred to a nylon membrane using the Semi-Dry Electrophoretic Transfer Cell system (Bio-Rad; Hercules, CA, USA). The target proteins were detected by rabbit polyclonal antibodies against Csa1, Cas1 and Csa3a, and then bound by horseradish peroxidase-labeled goat anti-rabbit antibody (Beyotime, Beijing, China). Bands were visualized by chemiluminescent detection using the clarity Western ECL substrate (Bio-Rad; Hercules, CA, USA) and the MF-Chemibis 3.2 imaging device (DNR; Jerusalem, Israel).

### PCR amplification, cloning and sequencing of leader proximal CRISPR region

Wild-type *S. islandicus* E233S, E233S carrying an empty expression vector and a *csa3a*-overexpression strain were cultured in 10 ml of ACVy medium at 78°C for 10 days. Samples of each culture (0.1 ml) were taken every 24 h and cells were used for PCR template. The leader proximal regions of two CRISPR loci were amplified by PCR using Taq polymerase and forward primer CRISPR-F and reverse primer CRISPR2S5-R for locus 2; and forward primer CRISPR-F and reverse primer CRISPR1S5-R for locus 1. PCR products were separated on 2% agarose gel and visualized by ethidium bromide staining. Bands larger than those of the wild-type control sample were excised from the gel and purified by Axygen DNA extraction kit. Purified PCR products were cloned into T-vector (Takara, Dalian, China) following the manufacturer's instruction, then the ligation products were transformed in to *E. coli* DH5α. Plasmids from single colonies were purified and sequenced at Invitrogen (Shanghai, China).

## RESULTS

### Co-transcription of the *csa1, cas1, cas2* and *cas4* genes in *S. islandicus*

The *S. islandicus* REY15A genome encodes a single gene cassette containing the *csa1* (SiRe_0760), *cas1* (SiRe_0761), *cas2* (SiRe_0762) and *cas4* (SiRe_0763) genes, in which *cas1* and *cas2* have been implicated in spacer acquisition in this archaeon (Figure 1A). To investigate the function of these *cas* genes and the regulation of their gene expression, total RNA was prepared and the co-transcription of these genes was studied. A 1016-bp DNA fragment was amplified using primers *csa1*qF and *cas1*qR from the first-strand cDNA, which was synthesized using *cas1*qR as a primer from total RNA (Figure 1B). This result indicated that the *csa1* and *cas1* genes were co-transcribed under the control of the *csa1* promoter. However, no PCR products were amplified using *csa1*qF with *cas2*qR or *cas4*qR from the first-strand cDNAs that were synthesized using primers *cas2*qR and *cas4*qR (Figure 1B). This result showed that the *cas2* and *cas4* genes were not co-transcribed with *csa1*. Interestingly, three DNA fragments (1151bp, 1418 bp and 376 bp) were amplified from the first-strand cDNAs using primer pairs *cas1*qF-*cas2*qR, *cas1*qF-*cas4*qR and *cas2*qF-*cas4*qR, which were synthesized using the oligonucleotides *cas2*qR and *cas4*qR from total RNA. These results strongly indicated that *cas1, cas2* and *cas4* were cotranscribed. This cotranscription results are consistent with promoters of *csa1* and *cas1* controlling the transcription of this operon. Thus, the operon was named adaptation-associated *cas* operon (a*cas* operon) according to the aCas complex annotated by Garrett *et al*. (46).

### Csa3a bound both *csa1* and *cas1* promoters *in vivo*

**Figure 2.**
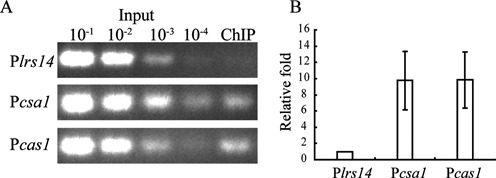
*In vivo* binding of Csa3a to the *csa1* and *cas1* promoters. (**A**) Chromatin immunoprecipitation (ChIP) assay using antiserum against Csa3a. Input samples were 10^−1^, 10^−2^, 10^−3^ and 10^−4^ of the input sample amounts described in the ‘Materials and Methods’ section. DNA recovered from immunoprecipitates was amplified with primers specific for *csa1* and *cas1* promoters, or the *lrs14* promoter as a control. (**B**) The ChIP-qPCR result revealed that *csa1* and *cas1* promoter sequences in the recovered DNA were ∼10-fold relative to the *lrs14* promoter sequence.

The *csa3a* gene (SiRe_0764), encoding a putative transcriptional regulator, is adjacent to the a*cas* operon but in an inverse orientation in the *S. islandicus* REY15A chromosome (Figure 1A). This is consistent with Csa3a functioning as a transcriptional regulator for the a*cas* genes. Therefore, a ChIP assay was conducted to test for an interaction between Csa3a and a*cas* promoters. The results in Figure 2A demonstrate that Csa3a bound specifically to the *csa1* and *cas1* promoters. Moreover, no PCR product for the promoter of *lrs14*, a transcriptional regulator gene, was found in the negative control experiment. The recovered DNA sample from the ChIP assay was quantified by real-time PCR for the *csa1, cas1* and *lrs14* promoters. The recovered DNA contained ∼10-fold more DNA of both the *csa1* and *cas1* promoters than of the control *lrs14* promoter (Figure 2B). The results confirmed that Csa3a bound to both *csa1* and *cas1* promoters, suggesting that Csa3a is involved in transcriptional regulation of the a*cas* operon.

### Localizing the Csa3a-binding sequence in the *csa1* promoter

**Figure 3.**
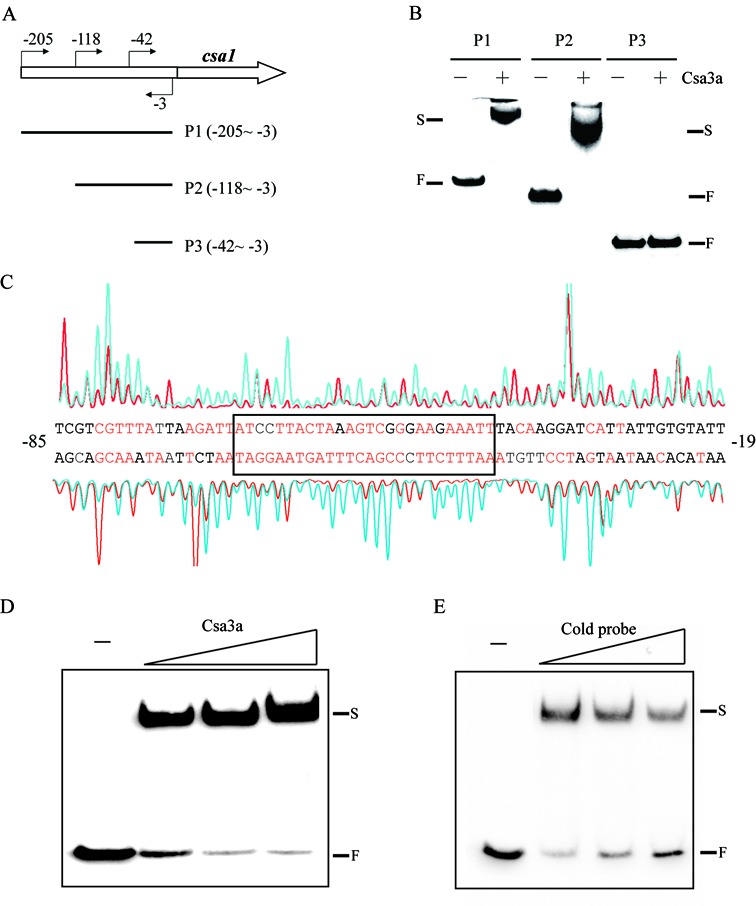
*In vitro* binding of the *csa1* promoter by Csa3a. (**A**) Schematic diagram of the *csa1* promoter and the DNA probes used for EMSA. The locations of primers used for amplifying the probes are indicated. (**B**) EMSA experiments using three truncated probes in the absence or presence of Csa3a (1.6 pmol/μl). F: free labeled probe; S: shifted band. (**C**) DNase I footprinting assay with coding (HEX-labeled, peaks above the promoter sequences) and non-coding (FAM-labeled, peaks below the promoter sequence) strands of the DNA fragments containing the *csa1* promoter in the presence (red peaks) and absence (blue peaks) of Csa3a. The protected nucleotides (relative to the translational start codon) are shown in red and the minimal region is boxed. (**D**) EMSA experiment using the sequence of the boxed region indicated in (C) as a probe with increasing amounts of Csa3a (0.4, 0.8 and 1.6 pmol/μl). (**E**) EMSA experiment in the presence of Csa3a (0.4 pmol/μl) using the same probe as in (D) with increasing amounts of unlabeled nucleotides covering the *csa1* promoter (molar ratio = 1: 1, 1: 4 and 1: 8).

To study the regulation of the *csa1* promoter by Csa3a, the promoter was used as the probe in an EMSA experiment. In an archaeal promoter, the hexameric TATA box is centerd at −26/−27 with respect to the transcription start site and BRE (transcription factor B recognition element) located immediately upstream of the TATA box. Thus, the promoter fragment P1 (positions −205 to −4 relative to the translational start codon) and two 5′-truncated elements P2 (positions −118 to −4) and P3 (positions −42 to −4) were used in the EMSA experiment to determine the Csa3a-binding site (Figure 3A). Both P1 and P2 DNA fragments produced strong band shifts with Csa3a on the gel, whereas no such retarded band was observed when the P3 fragment was incubated with Csa3a (Figure 3B). We inferred that the binding site for Csa3a is located between positions −118 and −42 on the *csa1* promoter relative to the translational start codon.

### Identification of the minimal region of Csa3a binding in the *csa1* promoter

To determine the precise binding region of Csa3a on the *csa1* promoter, a footprinting assay was carried out and the region from −81 to −42 of both strands of the promoter sequence was shown to be protected by Csa3a (Figure 3C). This sequence exhibited an imperfect inverted repeat close to the BRE and TATA box (Supplementary Figure S2). The 25-bp fragment was then used as a probe in the EMSA experiment to test further the DNA binding capacity of Csa3a. The results show that increasing the amount of Csa3a protein enhanced the signal of the retarded bands (Figure 3D) and, moreover, that increasing the amount of the unlabeled specific probe selectively reduced the signal of the retarded bands (Figure 3E). Therefore these results define the minimal region for Csa3a binding on the *csa1* promoter.

### Identification of two DNA elements essential for Csa3a binding in the *cas1* promoter and adjacent sequence

**Figure 4.**
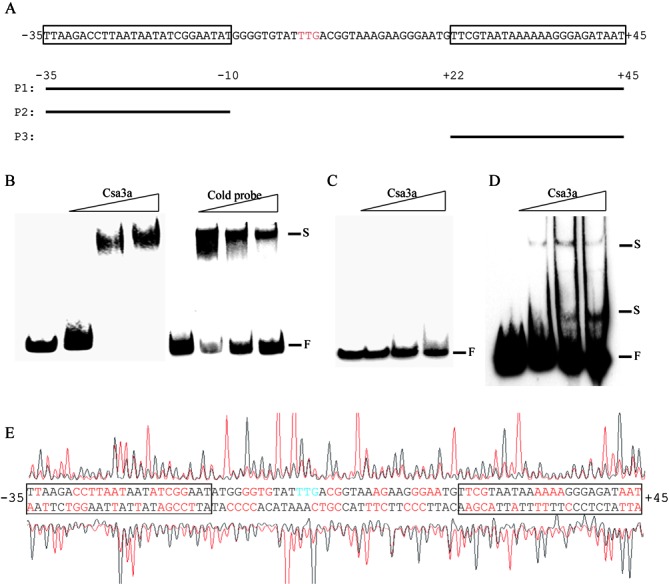
Identification of binding site of Csa3a on the *cas1* promoter and *cas1* coding sequence. (**A**) Schematic diagram of the *cas1* promoter and the DNA probes used for EMSA. The translational start codon (TTG) is indicated in red. The locations of three probes relative to the translational start codon are indicated. Two regions similar to the minimal binding site of Csa3a on the *csa1* promoter are boxed. (**B**) EMSA experiments using biotin labeled P1 as a probe with increasing amounts of Csa3a (0.4, 0.8 and 1.6 pmol/μl) or an unlabeled P1 cold probe (molar ratio = 1: 1, 1:4 and 1:8). (**C** and **D**) EMSA experiments using biotin labeled P2 or P3 as a probe with increasing amounts of Csa3a (2.4, 4.8, 6 pmol/μl). F: free labeled probe; S: shifted band. (**E**) DNase I footprinting assay with coding (HEX-labeled, peaks above the promoter sequences) and non-coding (FAM-labeled, peaks below the promoter sequence) strands of the DNA fragments containing the *cas1* promoter in the presence (red peaks) and absence (black peaks) of Csa3a. The protected nucleotides (relative to the translational start codon) are shown in red and the regions similar to the Csa3a-binding site on the *csa1* promoter are boxed.

Since Csa3a binds both *csa1* and *cas1* promoter sequences *in vivo* and *csa1* and *cas1* are transcribed separately, we decide to analyze the interaction between *cas1* promoter and Csa3a. In the region between positions −35 to +45 of the *cas1* promoter (relative to the translational start codon, TTG), and its adjacent sequence, two DNA fragments were found by alignment that were similar in sequence to the minimal sequence required for Csa3a binding on the *csa1* promoter (Figure 4A). Thus, a full-length sequence (P1, −35 to +45), an upstream DNA element (P2, −35 to −10) and a downstream element (P3, +1 to +45) were used as probes in the EMSA experiment to test for binding of Csa3a (Figure 4A). At increasing concentrations of Csa3a, the signals of the P1 retarded bands became stronger, while increasing amounts of the cold probe reduced the signals of the retarded bands (Figure 4B). These results indicated that Csa3a specifically bound to the *cas1* promoter and its adjacent sequence. However, the EMSA results showed that increasing the amount of Csa3a did not retard the P2 fragment (Figure 4C) and that Csa3a was only weakly bound to the P3 fragment (Figure 4D). Csa3a weakly retarded P4 and P5 (P4: P1 with deletion of P2 element; P5: P1 with deletion of P3 element), but did not retard the internal sequence itself (Supplementary Figure S3). Thus, in contrast to the Csa3a-binding to the *csa1* promoter, efficient binding of Csa3a to the *cas1* promoter requires both P2 and P3 sites and the internal element between P2 and P3.

A footprinting assay using a fragment covering positions −143 to +97 of the *cas1* promoter sequence, was performed to determine the precise Csa3a-binding sites. Csa3a protected a large region of the fragment extending from position −35 to +45 (Figure 4E). The protected region contained two elements that are highly similar to the Csa3a-protected sequence on the *csa1* promoter (Figure 4E and Supplementary Figure S2). It was revealed that neither site alone was sufficient for Csa3a binding (Figure 4C and D) but that the two regions appeared to act cooperatively in facilitating Csa3a recognition.

### Overexpression of *csa3a*-activated transcription of a*cas* genes

**Figure 5.**
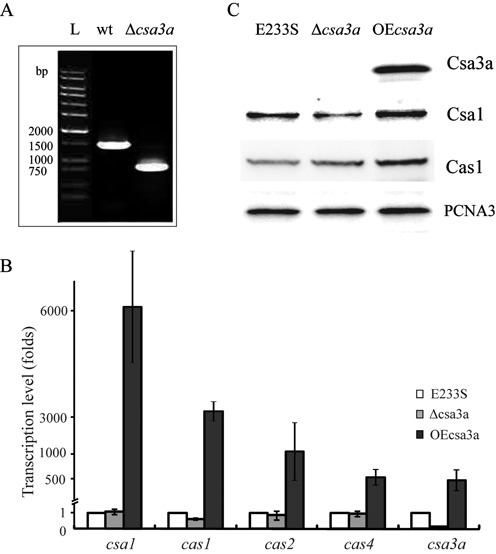
Effect of Csa3a on the expression of ac*as* genes. (**A**) PCR verification of the *csa3a*-deletion mutant. PCR products obtained using specific primers located upstream and downstream of *csa3a* gene from genomic DNAs of *Sulfolobus islandicus* E233S (wild-type) and the *csa3a*-deletion strain (Δ*csa3a*). A DNA ladder was run in lane L as a size marker. (**B**) The relative transcription levels of *csa3a, csa1, cas1, cas2* and *cas4* in *S. islandicus csa3a-*overexpression (OE*csa3a*) and *csa3a*-deletion (Δ*csa3a*) strains after normalization to the level in *S. islandicus* E233S (wild-type) carrying empty vector pSeSD. (**C**) Western blot analysis of Csa3a, Csa1 and Cas1 protein expression levels in *S. islandicus* E233S (wild-type), *csa3a* overexpression (OE*csa3a*) and *csa3a*-deletion (Δ*csa3a*) strains. Antiserum against PCNA3 was used as a loading control.

Although the specific *in vitro* and *in vivo* binding of Csa3a to the *csa1* or *cas1* promoter was confirmed, the specific mechanism of transcriptional regulation of the a*cas* operon remained unclear. Therefore, we constructed a *csa3a* gene-deletion mutant (Δ*csa3a*, Figure 5A) and a *csa3a-*overexpression (OE*csa3a*) strain (Figure 5A). Expression of the *csa3a, csa1, cas1, cas2* and *cas4* genes was then tested in the wild-type (carrying the empty vector pSeSD), Δ*csa3a* and OE*csa3a* strains. *Sulfolobus* strains were grown in ACVy medium to OD_600_ = 0.2 and then cells were harvested and total RNA and crude protein samples were prepared. The transcripts of the a*cas* genes were determined by RT-qPCR using mRNA of the *albA* gene as an internal control(45). The *csa3a* transcription level in OE*csa3a* strain increased by 489(±105)-fold compared with the wild-type strain and no cDNA was detected in the Δ*csa3a* strain. The transcription levels of a*cas* genes were reduced by 1.00(±0.18)-, 0.62(±0.08)-, 0.84(±0.27)- and 0.94(±0.16)-fold in the Δ*csa3a* strain and increased by 6279(±1583)-, 3331(±273)-, 1291(±481)- and 525(±79)-fold in the OE*csa3a* strain, compared with expression in the wild-type strain (Figure 5B). Csa3a was detected neither in wild-type nor in Δ*csa3a* strains in a western blot assay, while a strong Csa3a protein band from the OE*csa3a* strain was visualized on the membrane (Figure 5C). Csa1 and Cas1 proteins showed elevated expression in the OE*csa3a* strain and the expression levels in Δ*csa3a* strain were similar to that in wild-type strain (Figure 5C). This result revealed that Csa3a acts as a transcriptional activator for the a*cas* operon. However, Cas2 and Cas4 proteins were not detected in the wild-type, Δ*csa3a* or OE*csa3a* strains by western blotting consistent with low protein levels in the cells. PCNA3 was used as the internal control in western blotting and its expression showed no difference among the wild-type, Δ*csa3a* and OE*csa3a* strains. These results clearly indicated that the Csa3a regulator acts as a transcriptional activator for a*cas* genes in *S. islandicus*; however, translation of the a*cas* mRNA may be controlled by an unknown mechanism.

### Activation of *de novo* spacer acquisition by overexpression of *csa3a*

**Figure 6.**
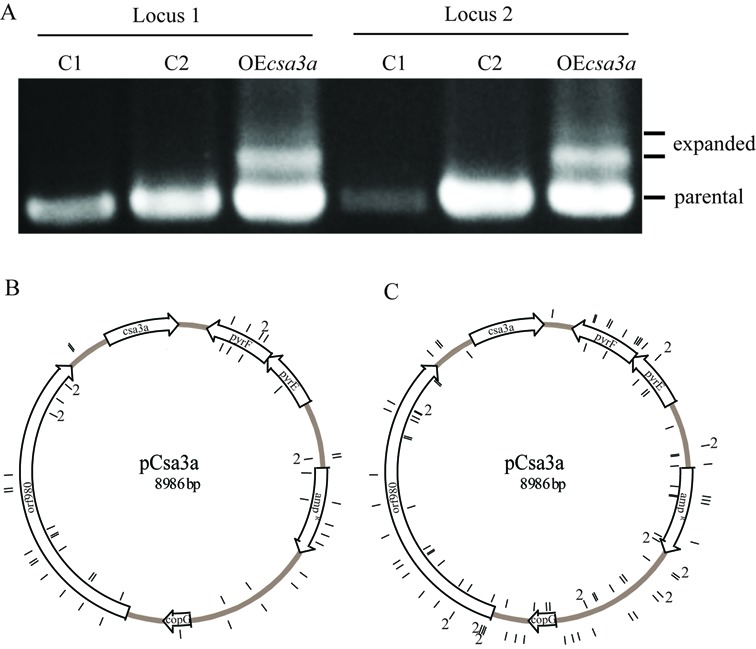
Csa3a triggers CRISPR spacer acquisition in *Sulfolobus*. (**A**) Spacer acquisition was detected by PCR for both loci in *csa3a* overexpression strain. C1: *S. islandicus* E233S strain, C2: E233S carrying empty vector pSeSD, OE*csa3a*: E233S carrying *csa3a* overexpression plasmid. The *csa3a* expression plasmid is shown with individual genes indicated (**B** and **C**). Locations of the protospacers corresponding to newly acquired plasmid-derived spacers on locus 1 (B) and locus 2 (C) are shown in the inner (forward strand) and outer (reverse strand) circles.

Wild-type (*S. islandicus* E233S), E233S carrying an empty expression vector and *csa3a*-overexpression strains, were cultured in ACVy medium and the latter strain showed retarded growth from the beginning of inoculation (data not shown). Larger DNA fragments amplified between the leader sequence and spacer 5 of loci 1 and 2 were observed after 1 day of incubation in the medium (Figure 6A). Two larger fragments were produced by PCR, with the strong band containing one *de novo* spacer and the weak band carrying two spacers for both CRISPR loci (Figure 6A). No expanded bands were detected from E233S and E233S carrying empty vector.

DNA from the larger PCR products was cloned and transformed into *E. coli* and DNA from single colonies was sequenced. This revealed large number of *de novo* spacer sequences in each locus (all protospacers were summarized in Supplementary Table S2). For locus 1, 52 unique spacers were acquired with 96.1% of the spacers from the *csa3a* expression plasmid and 3.9% from genomic DNA. For locus 2, 108 unique spacers were acquired with 91.7% of the spacers from the plasmid DNA and the remainder from genomic DNA. Most of the colonies carried only one new acquired unique spacer adjacent to leader sequence and 15 colonies exhibited two and one colony showed four *de novo* spacers (Table 1). The locations of these new spacers did not show significant bias for protein genes or intergenic sequences. However, no spacer was found to originate from the *csa3a* gene on the expression plasmid, which may reflect that strains acquired spacers from *csa3a* will target another copy of this gene on their own genome, thus no survival strains carrying new spacer from *csa3a* gene sequence were detected (Figure 6B and C)(47). Total 11 spacers were found to originate from the host genome. Two protospacers matched to SiRe_0056 gene (inosine/uridine-preferring nucleoside hydrolase gene) and two matched to SiRe_1054 gene (adenylate kinase related protein gene). Each one protospacer matched to SiRe_0560 (major facilitator superfamily MFS_1), SiRe_0654 (4Fe-4S ferredoxin iron-sulfur binding domain protein gene), SiRe_1500, SiRe_1555 (transcription initiation factor TFIIB gene), SiRe_2286 (major facilitator superfamily MFS_1), SiRe_2332 (malate dehydrogenase) and SiRe_2393 (mechanosensitive ion channel), respectively. There was no evidence for biased strand directionality for the acquired spacers of both loci (Figure 6B and C; Table 1). Moreover, the PAM sequence CCN was conserved in adjacent to protospacer sequences, and −1 site of PAM was biased to A (Table 1), which was consistent with previous study in *Sulfolobus* (21).

**Table 1.** Analysis of the protospacers

	Locus 1	Locus 2
Protospacer location		
Total	52	108
Plasmid	50 (96.1%)	99 (91.7%)
Genomic DNA	2 (3.9%)	9 (8.3%)
Forward strand	28 (53.8%)	60 (55.6%)
Reverse strand	24 (46.2%)	48 (44.4%)
Within protein gene	40 (76.9%)	72 (66.7%)
Intergenic	12 (23.1%)	36 (33.3%)
PAM sequences		
CCN	35 (67.3%)	80 (74.1%)
CCA	12 (23.1%)	35 (32.4%)
CCT	12 (23.1%)	20 (18.5%)
CCG	6 (11.5%)	15 (13.9%)
CCC	5 (9.6%)	10 (9.3%)
No PAM	17 (32.7%)	28 (25.9%)
Single and multiple spacer insertion		
Single	42	84
Two	3	12
Four	1	

A total of 160 unique spacers inserted into loci 1 and 2 were sequenced and analyzed. The numbers of protospacers matching the expression plasmid and genomic DNA, as well as the protospacers carrying PAM sequences (5′-PAM-protospacer-3′), are given. Percentages are included in brackets.

## DISCUSSION

In these experiments, we investigated the transcriptional regulation of the a*cas* operon, containing *cas1* and *cas2* genes, by the CRISPR-Cas-specific regulator Csa3a both *in vivo* and *in vitro*. The *csa3* gene is also named as *csx1* and is divided into subtype III-U (2). The *csx1* genes are often found physically linked to archaeal *cmr* gene cassettes, however, it also appears in Type I modules, which suggests a broader role for this gene (37,48). Csa3a protein is predicted to have an N-terminal domain of unknown function fused to a C-terminal MarR-like winged helix-turn-helix domain which suggests that it functions as a transcriptional regulator. The crystal structure of Csa3 from *S. solfataricus* P2 (Mw = 27.8 kDa) which is closely related to Csa3a of *S. islandicus* REY15A (66% identity) has recently been solved (49). Our results demonstrated that overexpression of *csa3a* triggered *de novo* spacer acquisition in the Type I-A CRISPR-Cas system of *Sulfolobus*. Moreover, it was shown that Csa3a bound to an imperfect palindromic sequence (TTCNTAACTAAANANGGNNNGAAA, Supplementary Figure S2A) upstream of the *csa1* promoter or two similar elements covering the BRE and TATA box of the *cas1* promoter and the translational start codon. Overexpression of *csa3a* greatly increased the transcriptional level, and moderately increased translational level, of a*cas* genes, demonstrating that Csa3a acted as a transcriptional activator. Csa3a may regulate transcription of other genes, and even CRISPR loci, however, this need to be further confirmed. The Csa3a-binding site on the *csa1* promoter represents a typical activation *cis*-element in archaea, located immediately upstream of core promoter elements (Supplementary Figure S2B) and the binding proteins recruit general transcription factors to activate transcription (50). The two Csa3a-binding sites on the *cas1* promoter covering the TATA box, BRE and translational start codon (Supplementary Figure S2B), presented an atypical mechanism of transcription activation. However, the *in vivo* data suggested that Csa3a activated the transcription of the *cas1* gene (Figure 5B and C). Our results are also consistent with the transcriptomic data of *S. islandicus* LAL 14/1, in which up-regulation of the a*cas* operon was accompanied by increased *csa3a* transcript levels after rudivirus infection (48). These results suggested that *csa3a* was transcribed at a low level before virus infection, while after infection elevated Csa3a levels may activate transcription of a*cas* genes. Indeed, Csa3a was not detected in the wild-type strain by western blotting and we also confirmed that the promoter strength of both *csa1* and *cas1* promoters was very low (∼10^−6^-fold) compared with that of the *araS* promoter (50) (Supplementary Figure S2C). Thus we infer that the low transcriptional level of *csa3a* gene caused a low expression activity of a*cas* operon.

**Figure 7.**
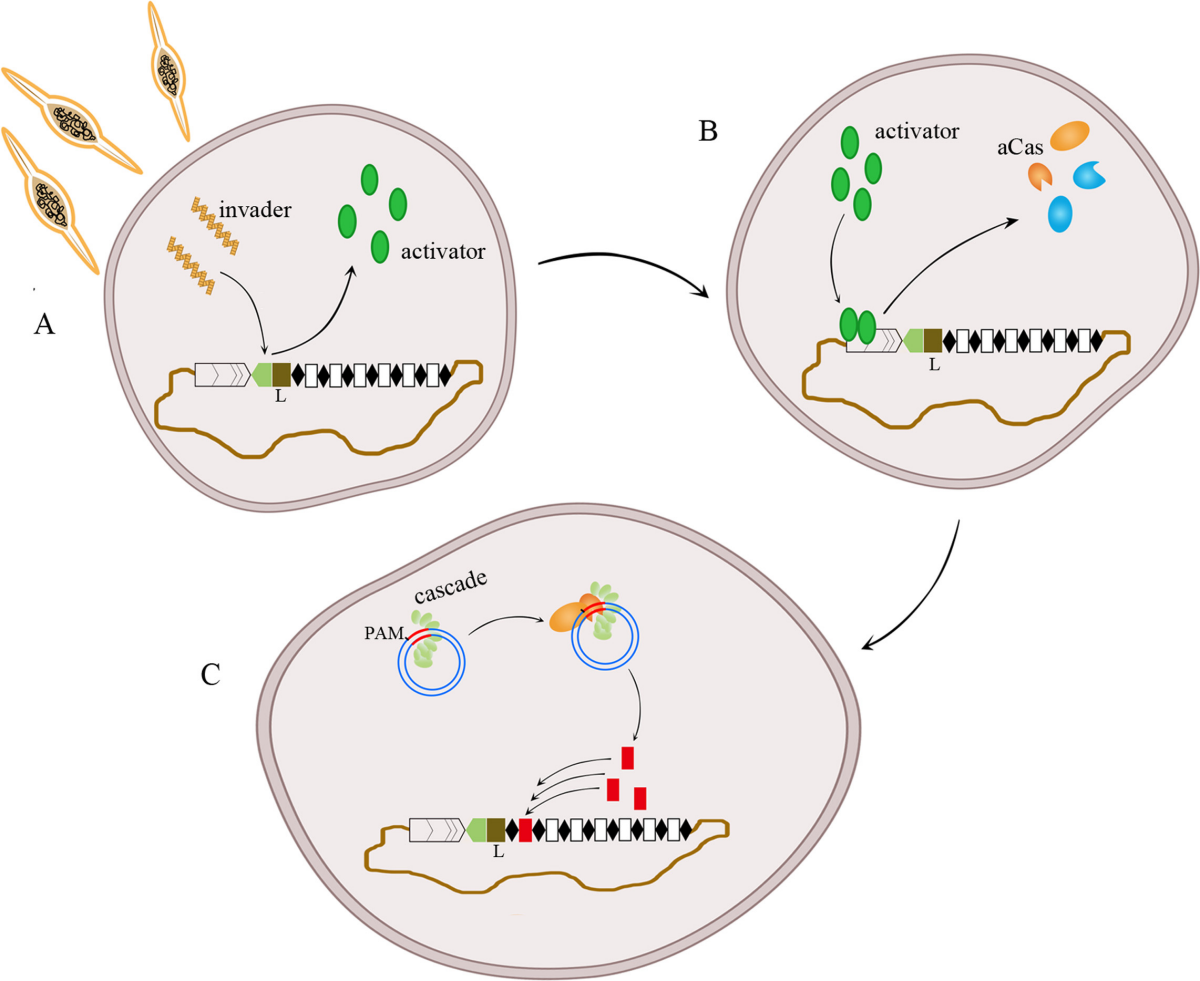
A CRISPR *de novo* spacer acquisition model that is transcriptional regulator-mediated and Cas1- + Cas2-dependent. (**A**) During infection with virus and other genetic mobile elements, a transcriptional regulator senses the invader and its expression is activated. (**B**) The regulator strongly activates the transcription of a*cas* genes, including *cas1* and *cas2*. (**C**) Cas1, Cas2 and other Cas proteins (Cascade and Cas3 are possible) together recognize PAM sequences of the protospacer in the invader DNA and *de novo* spacers are inserted into the CRISPR locus adjacent to the leader sequence.

It was previously reported that virus infection can trigger spacer acquisition. For example the monocaudavirus SMV1 induced spacer acquisition from coinfecting pMGB1 and STSV2 genomes in *Sulfolobus* (20,21) and priming spacer-mediated spacer acquisition was observed in *H. hispanica* (22), *E. coli* (16,25,29) and *P. atrosepticum* (24). It has also been reported that overexpression of Cas1 and Cas2 can facilitate spacer acquisition in *E. coli* (17,25). However, the basic mechanism by which the aCas complex is activated for adaptation remains unknown. In this study, overexpression of the *csa3a* gene on a plasmid strongly activated transcription of the a*cas* operon but the translational level of *acas* operon was increased to a lesser degree than the transcriptional level (Figure 5B). Most importantly, we observed that overexpression of *csa3a* gene triggered *de novo* spacer acquisition efficiently. Thus, we demonstrated that the transcription regulator Csa3a played a crucial role in this pathway and a model is proposed for this process in Figure 7: step 1: invasion of virus or other mobile genetic elements induces expression of the transcriptional regulator gene; step 2: the regulator largely activates transcription of the a*cas* operon, including *cas1* and *cas2*; step 3: Cas1, Cas2 and other Cas proteins forming the aCas complex take up new spacers from the invader DNA. The first step has been confirmed in *S. islandicus* LAL 14/1, that *csa3a* gene was induced in the late phase after virus infection. However, a*cas* genes were induced slightly in the early phase, which may suggest other factors but not Csa3a were also involved in a*cas* induction. Anyhow, no *de novo* spacer acquisition was observed for this strain after virus challenging (48). The increase in *csa3a* and a*cas* transcription was much higher in our study. This suggests that an induction threshold value of *csa3a* is required for efficient activation of a*cas* genes. Our model suggests further that the Cas1- and Cas2-dependent spacer acquisition process has an efficiency as high as the priming process, as has been reported previously in other archaea or bacterium (22,25). In our model, the *de novo* spacer acquisition process needs to be largely activated by a transcription regulator which may sense invading genetic elements.

Our results also indicate the greatly induced Cas1- and Cas2-dependent spacer acquisition process is dependent on the PAM sequence of the protospacers (Table 1) but it is not as strict as was observed for the SMV1 virus-induced spacer acquisition (51). Moreover, for an *E. coli* CRISPR-Cas minus strain it was reported that overexpression of *cas1* and *cas2* genes led to *de novo* spacer acquisition from protospacer with less conserved PAM sequence (17). Therefore, we infer that a Cas protein other than Cas1 and Cas2 is responsible for PAM recognition, and that an enhanced level of Cas1 and Cas2 proteins results in spacer acquisition from protospacers with less conserved PAM sequence.

## SUPPLEMENTARY DATA

Supplementary Data are available at NAR Online.

SUPPLEMENTARY DATA
