# Supplementary material for: Transcriptional regulator-mediated activation of adaptation genes triggers CRISPR de novo spacer acquisition
Source: Nucleic Acids Res. 2015 Jan 7;43(2):1044–55. doi: 10.1093/nar/gku1383 (PMC4333418; doi:10.1093/nar/gku1383)
Supplement: SUPPLEMENTARY DATA [file supp_43_2_1044__index.html]

Transcriptional regulator-mediated activation of adaptation genes triggers CRISPR de novo spacer acquisition — SUPPLEMENTARY DATA 

# Transcriptional regulator-mediated activation of adaptation genes triggers CRISPR *de novo* spacer acquisition

## SUPPLEMENTARY DATA

**Files in this Data Supplement:**

- SUPPLEMENTARY DATA
