# Supplementary material for: Transcriptional regulator-mediated activation of adaptation genes triggers CRISPR de novo spacer acquisition
Source: Nucleic Acids Res. 2015 Jan 7;43(2):1044–55. doi: 10.1093/nar/gku1383 (PMC4333418; doi:10.1093/nar/gku1383)
Supplement: SUPPLEMENTARY DATA [file supp_gku1383_nar-03161-h-2014-File010.docx]

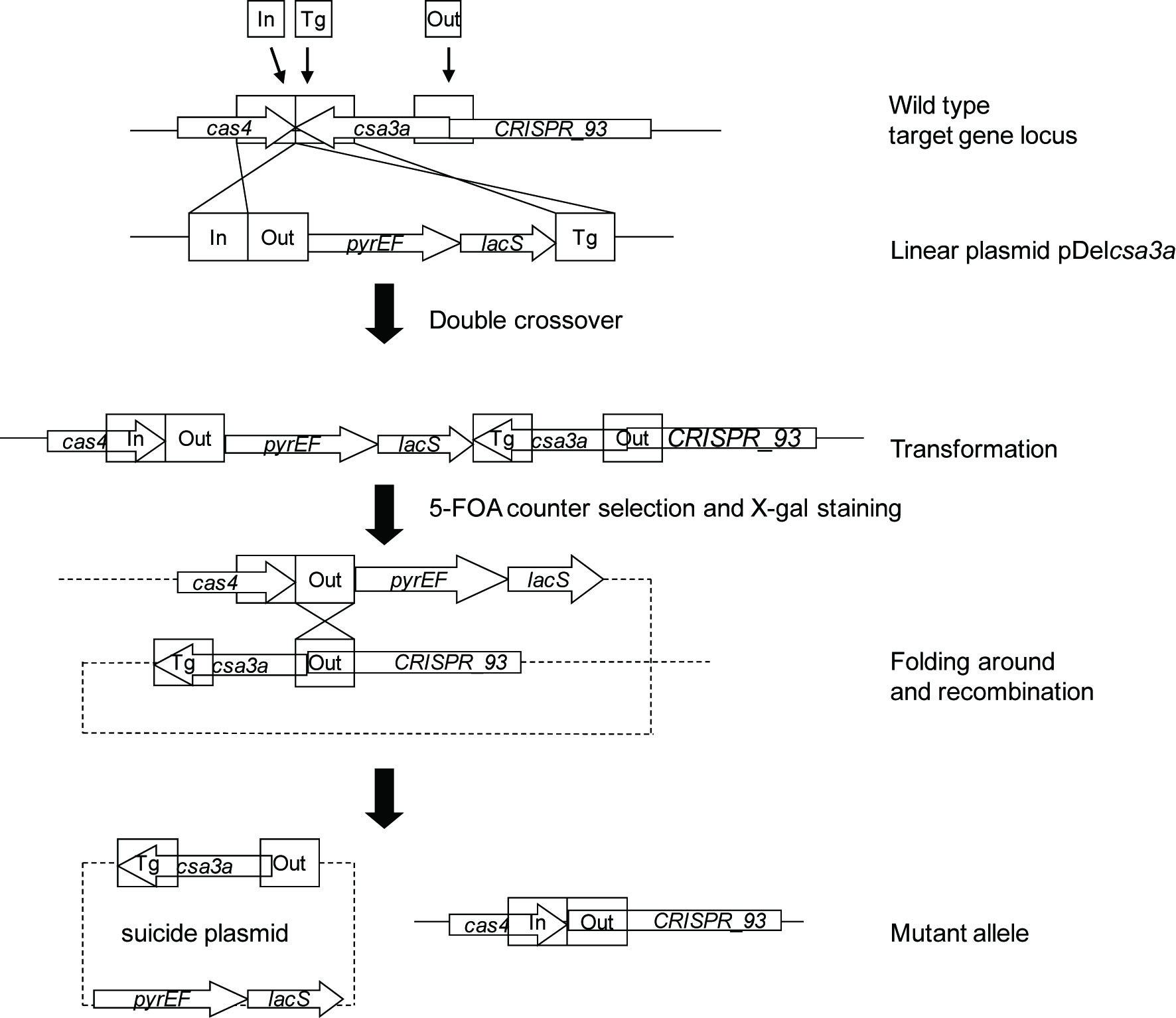


Figure S1. Flow chart of unmarked deletion of *csa3a* gene in *S. islandicus* E233S. Locations of the three homologous sequences, In-, Tg- and Out-arm, are indicated on the wild-type target gene locus in *S. islandicus* E233S chromosome. After transformation, double-crossover recombination between the linearized pDel*csa3a* plasmid and the host chromosome at the Tg-arm and the In-arm leads to the *pyrEF-lacS* cassette insertion at the target gene locus. Recombination between the two Out-arms loops out the target gene and *pyrEF-lacS* cassette, yielding *csa3a* deletion mutant that is counter-selectable on a plate containing 5-FOA.


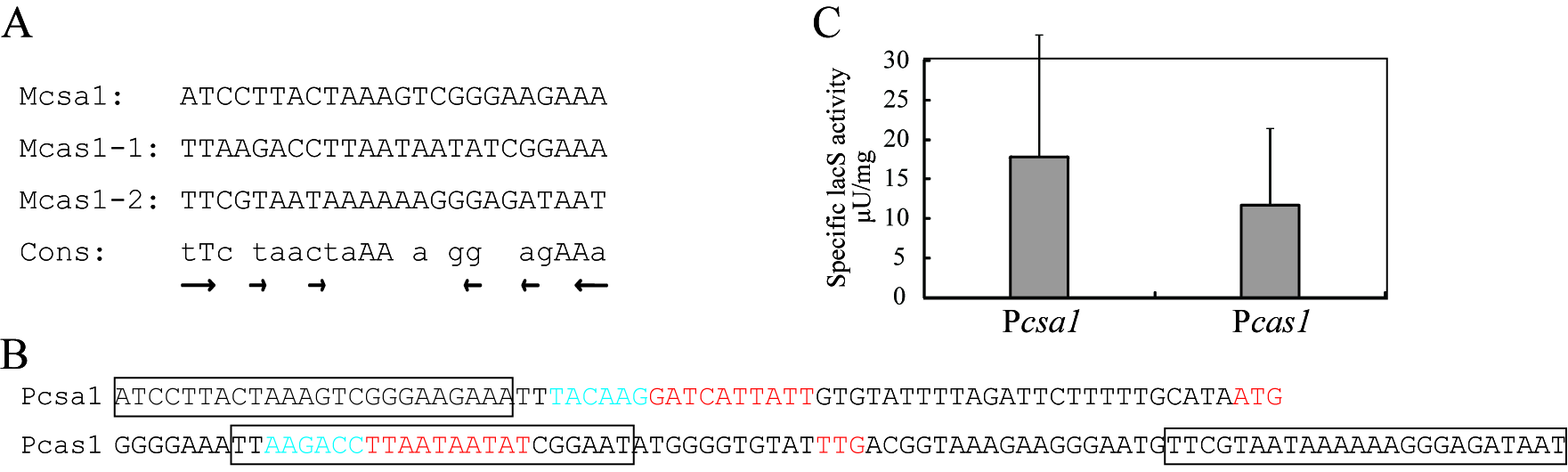


Figure S2. The promoter elements and activities of the *csa1* and *cas1* promoters. (A) The alignment of Csa3a-binding sites and the proposed consensus site. The imperfect inverted repeat is indicated by arrows. (B) Schematic diagram of *csa1* and *cas1* promoters used to control the transcription of the reporter gene *lacS*. Csa3a-binding sites are boxed and their position related to translation start codon are indicated; putative TATA box and the BRE are shown in red and blue, respectively. The translational start codons are indicated. (C) Specific β-galactosidase (lacS) activity of *csa1* and *cas1* promoters.


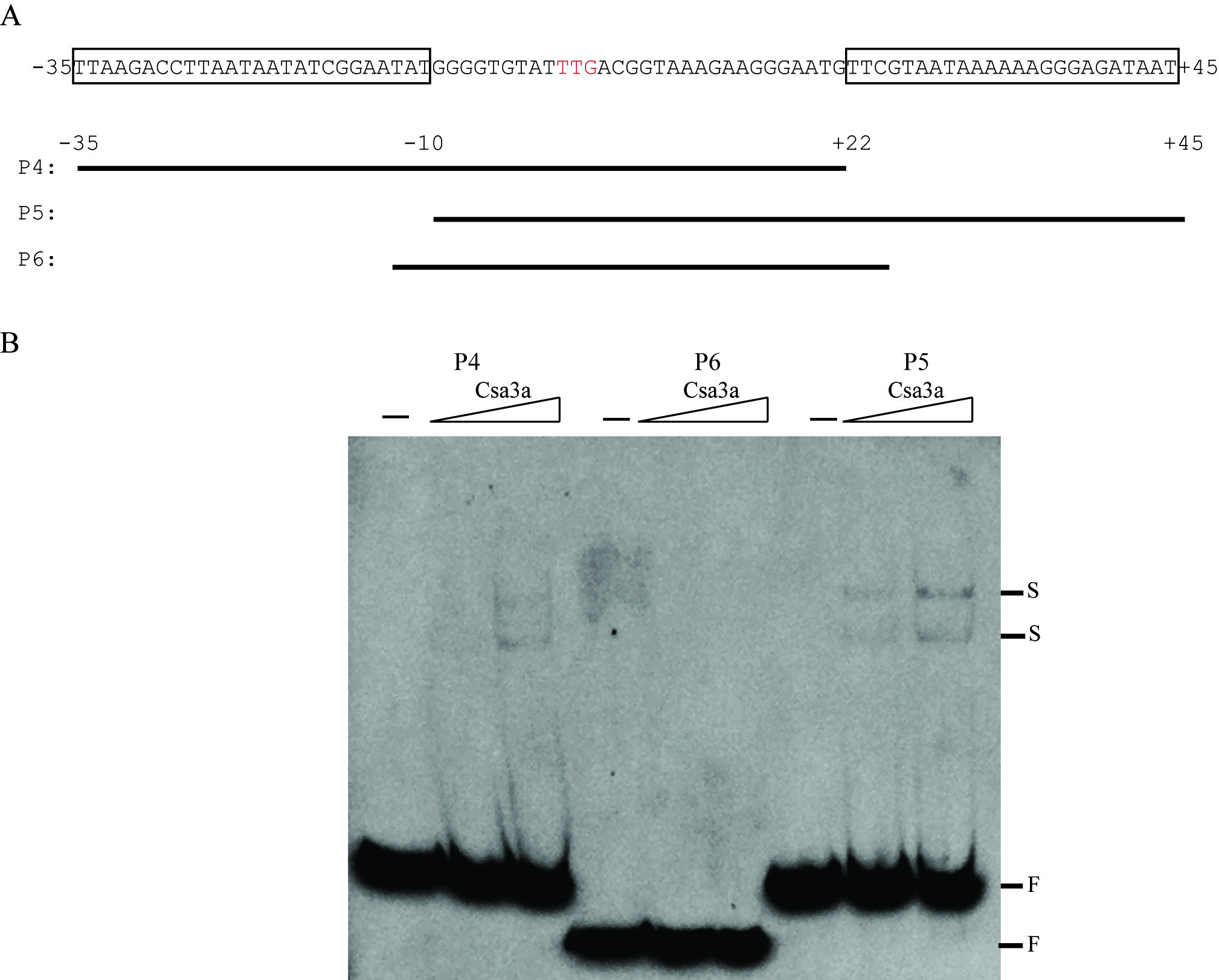


**Figure S3. Identification of binding site of Csa3a on the *cas1* promoter and *cas1* coding sequence.** (A) Schematic diagram of the *cas1* promoter and the DNA probes used for EMSA. The translational start codon (TTG) is indicated in red. The locations of three probes relative to the translational start codon are indicated. Two regions similar to the minimal binding site of Csa3a on the *csa1* promoter are boxed. (B) EMSA experiments using biotin labelled P4, P5 and P6 as probes with increasing amounts of Csa3a (0.8 and 1.6 pmol/μL). F: free labelled probe; S: shifted band.

Table S1 primers used in this study

| Primers | Sequence (5′ to 3′) |
| --- | --- |
| Primer for RT-PCR and cloning | |
| *csa3a*-Lfwd-KpnI | AAGGTACCCGGGCATCCTTAGAAGAAGATA |
| *csa3a*-Lrev-MluI, | AAACGCGTAAATGAAATTCAATGATTTTAA |
| *csa3a*-Rfwd-MluI | CCACGCGTACAAAGTATGACTTCATCATTATA |
| *csa3a*-Rrev-BamHI | AAGGATCCTCTAGAGGATCCATTAGCACTGGT |
| *csa3a*-Gfwd-SalI | AAAAGTCGACTTATTTATTAAAATCATTGAA |
| *csa3a*-Grev-PstI | AACTGCAGTGGTGGTAGTAGAGTAGTATC |
| M-F-BamHI | GCGGGATCCAATCCTATCACATCAGCACCT |
| M-R-SalI | GCGGTCGACTTAGTGCCTTAATGGCTTTAC |
| *csa3a*F-NdeI | TATATATACATATGAAGTCATACTTTGTAACTTT |
| *csa3a*R-NotI | TTATGCGGCCGCTTGAATTTCATTTTCTATAT |
| *csa1*F-NdeI | GGGAATTCCATATGTATAATGTGAGGTCGC |
| *csa1*R-SalI | ACGCGTCGACCTGTAATAAGGGGTGA |
| *cas1*F- NdeI | GGGAATTCCATATGACGGTAAAGAAGGGAATG |
| *cas1*R- SalI | ACGCGTCGACGATTTAAACTAGTTTTGTAG |
| *cas2*F- NdeI | GGGAATTCCATATGAAAATAATTGTAGTTTATG |
| *cas2*R- SalI | ACGCGTCGACGGAGAATGGTTAGTGTGA |
| *cas3*F-NdeI | GGGAATTCCATATGTTGTCTTTAGCTGAC |
| *cas3*R-SalI | ACGCGTCGACATACACACCACCTAT |
| *cas4*F- NdeI | GGGAATTCCATATGGTTAGTGTGACCGATTTAAAG |
| *cas4*R- SalI | ACGCGTCGACCTTATCGGTAATCCTTTGA |
| *cas6*F-NdeI | GGGAATTCCATATGCCATTAATTTTCAAGAT |
| *cas6*R-SalI | ACGCGTCGACACCTTTAAAGTCTGAGG |
| P*csa1*RepF-SphI | ACATGCATGCAAGATTATCCTTACTAAAGTCGG |
| P*csa1*RepR-NdeI | GGAATTCCATATGTATGCAAAAAGAATCTAAAATACA |
| P*csa1*dRepF | CGAAATTTACAAGGATCATTATTGTGTATTTTAGATTCTTTTTGCATACA |
| P*csa1*dRepR | TATGTATGCAAAAAGAATCTAAAATACACAATAATGATCCTTGTAAATTTCGCATG |
| P*cas1*RepF-SphI | ACATGCATGCGGGGAAATTAAGACCTTAA |
| P*cas1*RepR-NdeI | GGAATTCCATATGATTATCTCCCTTTTTTATTACG |
| P*cas1*dRepR-NdeI | GGAATTCCATATGCAAATACACCCCATATTCC |
| Primers for RT-qPCR and ChIP qPCR | |
| *csa1*qF | AGACAGGTTAGGAGGCTTCAC |
| *csa1*qR | CAGATACACATCCCTTTTAG |
| *cas1*qF | TTTCCCCTTCTGAGGTTGAC |
| *cas1*qR | ACACCAATGCCGTGACTTAC |
| *cas2*qF | ATAGATAGTCAGCGAGTCAA |
| *cas2*qR | TTCTTTCCCAATCCCTTATG |
| *cas4*qF | AAATGACCTACCAGATCCCA |
| *cas4*qR | GCTACAATGTCCTTTGCTAA |
| *albA*qF | AGGCCGTAGATACTGTGGAAATAGT |
| *albA*qR | AAACTCTTGATTGTCTTCCGTCTTG |
| *csa3a*qF | GACTTCGCTATCCTAATCCG |
| *csa3a*qR | TATTCATAAGCCTCATTCCC |
| Primers for ChIP | |
| *csa1*F-ChIP | AAGATAATGAATTTAGGATAACAAC |
| *csa1*R-ChIP | GCAAAAAGAATCTAAAATACACAAT |
| *cas1*F-ChIP | GGGATGAGGTTATAGATATGGTA |
| *cas1*R-ChIP | AGGAAGATAAGACTAATATTTCG |
| *lrs14*F-ChIP | CTCAAGTTAGATCAGTATACATA |
| *lrs14*R-ChIP | AGCTAATTCATCTTCAGTCTTCG |
| Primers for EMSA | |
| *csa1*F*-*P1 | AAGATAATGAATTTAGGATAACAAC |
| *csa1*R*-*P1 | GCAAAAAGAATCTAAAATACACAAT |
| *csa1*F-P2 | TATATTTTCCATTATTCTTCATCAA |
| *csa1*R-P2 | GCAAAAAGAATCTAAAATACACAAT |
| *csa1*F-P3 | TTACAAGGATCATTATTGTGTATTTTAGATTCTTTTTGC |
| *csa1*R-P3 | GCAAAAAGAATCTAAAATACACAATAATGATCCTTGTAA |
| *cas1*F-P1 | TTAAGACCTTAATAATATCGGAATAT |
| *cas1*R-P1 | ATTATCTCCCTTTTTTATTACGAA |
| *cas1*F-P2 | TTAAGACCTTAATAATATCGGAATAT |
| *cas1*R-P2 | ATATTCCGATATTATTAAGGTCTTAA |
| *cas1*F-P3 | TTGACGGTAAAGAAGGGAATGTTCGTAATAAAAAAGGGAGATAAT |
| *cas1*R-P3 | ATTATCTCCCTTTTTTATTACGAACATTCCCTTCTTTACCGTCAA |
| Primers for footprinting | |
| *csa1*F | AAGATAATGAATTTAGGATAACAAC |
| *csa1*R | GCAAAAAGAATCTAAAATACACAAT |
| *cas1*F | GGGATGAGGTTATAGATATGGTA |
| *cas1*R | AGGAAGATAAGACTAATATTTCG |
| 6-HEX-M13F | TGTAAAACGACGGCCAGT |
| FAM-M13R | CAGGAAACAGCTATGACC |
| Primers for new spacer detection | |
| CRISPR-F | TTTCGTTTTGGGTTAGGTTG |
| CRISPR2S5-R | TCACTTTCGTAAAAACACTA |
| CRISPR1S5-R | TAGCTTATTCTCAGCGACAA |

Table S2 protospacers corresponding to newly acquired spacers

| Protospacers | Sequence (5′ to 3′) |
| --- | --- |
| Protospacer related to new spacers on locus 1 from plasmid | |
| 1 (+) | CCGGTGAGCGTGGGTCTCTCGGTATCATTGCAGCACTGGGGCCA |
| 2 (+) | CCAATTAAGAGCTCTCTAGTTTTATCCACTCCTAGATCTAA |
| 3 (-) | CCGATATCTGCAAGCTTAAAATCAACAATAATTTCCTCCACGT |
| 4 (+) | CCAAATCATAAAGAATGCCAAAAACAAGAAGACCAAAGAACTCAA  CCTATCGGTCCGTTTTTTGCGTAAGCCGTGTTAATATCTG |
| 5 (+) | CCATGCTTAACACGGCTTTCCAGGTTGTTTGTCAGGGTAAGA |
| 6 (+) | CCAAAGTCTTTGCCCCAAAATGGGACCGATACTTTTTACACA |
| 7 (-) | CCGCAGTGTTATCACTCATGGTTATGGCAGCACTGCATAATTC |
| 8 (+) | CCACAGGATTCCAAAGTCTTTGCCCCAAAATGGGACCGATACTTT |
| 9 (+) | CCGCTACCAGCGGTGGTTTGTTTGCCGGATCAAGAGCTACCA |
| 10 (+) | CGTTTTTTGCGTAAGCCGTGTTAATATCTGTATAATAATGGCGT |
| 11 (-,-,-,+) | CCCTTATCGGCATTCATAGCTTGTAACCTTAAAGAGCCTAT  CCATTTTGGGGCAAAGACTTTGGAATCCTGTGGTAAGAGTTGT  TCCCCGAAAAGTGCCACCTGACCTGGCGAAAGGGGGATGTGCTGCAAGG  CCTAGAAGAGATATTGAACTATAACAGCCTGGTGTTCATTAA |
| 12 (+) | ACCGCTCGCCGCAGCCGAACGACCGAGCGCAGCGAGTCAGTGAGC |
| 13 (-) | CCTCTACTTTCTTAACAACACCGTGTTGATCTTTGAAGA |
| 14 (-) | CCGCTATTCCTTTGCCGTTTAGCGTAAAGGCGGGATTAAACTTG |
| 15 (+) | CCAATTAAGAGCTCTCTAGTTTTATCCACTCCTAGATCTAA |
| 16 (+) | CCAGGTCAGGTGGCACTTTTCGGGGAAATGTGCGCGGAACCCCTA |
| 17 (+) | TGGCGAACTACTTACTCTAGCTTCCCGGCAACAATTAATAGACTGG  CCAAAATCCCTTAACGTGAGTTTTCGTTCCACTGAGCGTCAGA |
| 18 (+) | ATATAATAATAATGAATTAAAGATAGGTAGAGCTGATCTAAAGG |
| 19 (-) | TCGTCTCCCTTCGGGAAGCGTGGCGCTTTCTCATAGCTCACTTC |
| 20 (-) | CCGTTTTGCCCTTTAACTCATTATATTGACTTGTATACCA |
| 21 (-) | CCTAATTTTTCTAACTCTAACGCCCCATAGTTAATCGTGATCCCC |
| 22 (+) | CCTATAAAGGAATGTGCTATGAACGAGTTGGCGAAAGATAATCTTT |
| 23 (+) | CCTACAGCGTGAGCTATGAGAAAGCGCCACGCTTCCCGAAGGG |
| 24 (-) | CCAATTGGAAATCACGGGCTTTTTCGATTGCGGATCAATAG |
| 25 (+) | CCAGTCACAGAAAAGCATCTTACGGATGGCATGACAGTAAGAG |
| 26 (+) | CCTTCATGCTCTTAGGACCCCGTGATTCCGGCAAATCAACAT |
| 27 (-) | CCTTATGATATATTGTGAGAGAACTTCAGCCAATTCTTGTTTGAGT |
| 28 (-) | CCGTTTTGCCCTTTAACTCATTATATTGACTTGTATACCA |
| 29 (-) | CCTAATTTTTCTAACTCTAACGCCCCATAGTTAATCGTGATCCCC |
| 30 (-) | CCCACGCCCAACACGTAACTATTGTAACTCTGCAAATCCGCTAT |
| 31 (+) | AGCCACTTACCGATGAAGAGAAGAAGAGATTCTTAGAACAAATTGAAAAGGG |
| 32 (+) | CCCCGCCACAAAATTCAAGTTAGCCTTCATGCTCTTAGGACCCCG |
| 33 (-,+) | CCCAACTGATCTTCAGCATCTTTTACTTTCACCAGCGTTTCTG  CCTTGATCGTTGGGAACCGGAGCTGAATGAAGCCATACCAA |
| 34 (+) | CCAAATAACAGGTTTGCGGCGGGGTTTCTATTCCACAAACTTGT |
| 35 (-) | GCGCACATTTCCCCGAAAAGTGCCACCTGACCTGGCGAAAGGGGGA |
| 36 (-) | CCTAATAATCTTCTTAAGTGTTTCATCAATTGGTTCATAGATTCCCTT |
| 37 (-) | CTCAAAGGCGGTAATACGGTTATCCACAGAATCAGGGGATAACGCAGGAA |
| 38 (-) | CCTTATGATATATTGTGAGAGAACTTCAGCCAATTCTTGTTT |
| 39 (+) | GGCGGATGACAATATACCAGATCACAAGTATGATCCCGCCTTTA |
| 40 (-) | GCGCACATTTCCCCGAAAAGTGCCACCTGACCTGGCGAAAGGGGGA |
| 41 (-) | GTTATTCGCAGACTATATTATAAACGTGATAAGAGAGATAAGCCCTAA |
| 42 (+) | ATATAACTCATTTTCCATCTCTTTAAGAACTTGATATGAGAGAGG |
| 43 (+) | CGCCATAACTTCCACCTTGACTACCCATACCCGGAGAGAATT |
| 44 (-) | TAATCTCTCCGGGTATGGGTAGTCGAGGTGGAAGTTATGG |
| Protospacer related to new spacers on locus 1 from genome | |
| 1 (-) | AATATACTTTAGAGAAGACTCTGAGATAATAGATCTATCATAGG |
| 2 (+) | TGCCTCCTTAGCGTCTTGCGGCCATATCTCTGGAACTGGATTGG |
| Protospacer related to new spacers on locus 2 from plasmid | |
| 1 (+) | CCATAACTTCCACCTTGACTACCCATACCCGGAGAGACTAT |
| 2 (-) | CAAGCTGGGCTGTGTGCACGAACCCCCCGTTCAGCCCGACCGC |
| 3 (+) | CCCAAATAACAGGTTTGTGGCGGGGTTTCTATTCCACAAACT |
| 4 (-) | TAAAAACGTTATAAGAGAGATAAGCCCAAAAGGAATAGTAGTTGGAGGG |
| 5 (+) | CCTGATAAATGCTTCAATAATATTGAAAAAGGAAGAGTATGAG |
| 6 (-,-) | CCAAGTCATTCTGAGAATAGTGTATGCGGCGACCGAGTTGCT  CCACAGAATCAGGGGATAACGCAGGAAAGAACATGTGAGCAA |
| 7 (-) | CCTTATGATATATTGTGAGAGAACTTCAGCCAATTCTTGTTTGAGT |
| 8 (-) | CCCTTTAGATCAGCTCTACCTATCTTTAATTCATTATTATTA |
| 9 (+) | TCTTATTTATAGTTCTTAATGCTGTTAATGGATTCCCTGCATTAT |
| 10 (+) | CCTCAATATCTCATCATAAATTACAAAATACTCATACACACTTC |
| 11 (-) | CCAAATTTTCACCTTTTGCTATCGAAGTAGACTCCTCGGAT |
| 12 (-) | CATTCTCAGCGATCTGTCTATTTCGTTCATCCATAGTTGCCTGA |
| 13 (-) | TCATCGGTAAGTGGCTCATGGCTATATTTTTGCCAATTGGAAATCAC |
| 14 (+) | TTTCCCTATTGATCCGCAATCGAAAAAGCCCGTGATTTCCAATTGG |
| 15 (+) | CCCCAAAATGGGACCGATACTTTTTACACACGTTAATGAAAGCC |
| 16 (-) | CTAATTTTTCTAACTCTAACGCCCCATAGTTAATCGTGATCCCCACAAAAC |
| 17 (+) | CCATGCTTAACACGGCTTTCCAGGTTGTTTGTCAGGGTAAGA |
| 18 (+) | CCATTAACAGCATTAAGAACTATAAATAAGATTATCGAGGA |
| 19 (-) | CCCTGAACCGTGTCTGTATCAGACCTTATAGCCCACACTACCTC |
| 20 (+) | CCATTTTCCCTATTGATCCGCAATCGAAAAAGCCCGTGATTTCC |
| 21 (-) | CCAATGCTTAATCAGTGAGGCACCTATCTCAGCGATCTGTCTAT |
| 22 (+) | CCAAAATCCCTTAACGTGAGTTTTCGTTCCACTGAGCGTCAG |
| 23 (+) | CCCCTTCTTGTCTATCTATGATTACTAGTGCGTCTGAAACTTT  CCGTCGTTTTACAACGTCGTGACTGGGAAAACCCTGGCGTT |
| 24 (-) | CCGACCCTGCCGCTTACCGGATACCTGTCCGCCTTTCTCCCTT |
| 25 (-) | CCTGGTACGGCGTGTTGTTAAATACTTCAACACGGATCTTG |
| 26 (-) | CCTTGTTTTCACCTCAAATCATGGTGAATTATCCATTCCT |
| 27 (-) | CCAATGCTTAATCAGTGAGGCACCTATCTCAGCGATCTGTCTA |
| 28 (-) | CCGAATTTCTTTAGACACATCAGAAAATCTGGATCCAGATCTTTG |
| 29 (-) | CCATTAACAGCATTAAGAACTATAAATAAGATTATCGAGGATA |
| 30 (+) | CTAAATAAGATTTTTTTAGACCCTGACCCCGAAAACGTGAAAAA |
| 31 (-) | CCCTGGAAGCTCCCTCGTGCGCTCTCCTGTTCCGACCCTGCCGCT |
| 32 (+) | TGTGTTTCCTGTCCTATCGGTCCGTTTTTTGCGTAAGCCGTGTTAATATCTGT |
| 33 (-) | CCACCTGACCTGGCGAAAGGGGGATGTGCTGCAAGGCGATTAA |
| 34 (+) | CAAATACTGTTCTTCTAGTGTAGCCGTAGTTAGGCCACCACTTC |
| 35 (-,-) | CCAAGTCATTCTGAGAATAGTGTATGCGGCGACCGAGTTGCT  CCACAGAATCAGGGGATAACGCAGGAAAGAACATGTGAGCAA |
| 36 (+) | CCATATAACTCATTTTCCATCTCTTTAAGAACTTGATATGAGA |
| 37 (-) | CCCGAAAAGTGCCACCTGACCTGGCGAAAGGGGGATGTGCTGC |
| 38 (+) | TGATGATAACACGCCTCTTAGAATAGCTTTCGAAAACTGCACACTGG |
| 39 (-) | CCGCTATTCCTTTGCCGTTTAGCGTAAAGGCGGGATTAAACTTG |
| 40 (+) | CCAATTATCGGACCGATAGGACAGGAAATCATATATTTTTTAA |
| 41 (-) | CCTAACTTGTTTAACATGCATGCAGCACAATTGTACTTTAA |
| 42 (-) | CCCACACTACCTCAATATTCTTTCTATTAATGAACACCAGGC |
| 43 (+) | CCGTATTACCGCCTTTGAGTGAGCTGATACCGCTCGCCGCA |
| 44 (+) | CCGAATCTGATGTGGTAAAGATAGCACTGAAGAAGTTAGCA |
| 45 (-) | CCATAGAGAAAGCAACATTGGAGATTCTTAACGGTGGAGG |
| 46 (+) | CCGCATACACTATTCTCAGAATGACTTGGTTGAGTACTCACCAGT |
| 47 (-) | CCTTATGATATATTGTGAGAGAACTTCAGCCAATTCTTGTTTGA |
| 48 (+) | CCCTTAACGCCTATAAAGGAATGTGCTATGAACGAGTTGGC  ATTCAAAAACATAGTAATGGTGAATCAATTATAGGAATCTTCAGATGG |
| 49 (+) | CCAAAATCCCTTAACGTGAGTTTTCGTTCCACTGAGCGTCAGA |
| 50 (-) | CCTTTTTCAATATTATTGAAGCATTTATCAGGGTTATTGTCTCAT |
| 51 (+) | CCTTGACTACCCATACCCGGAGAGACTATGGTCATTTTTTCGAAGTC |
| 52 (+) | CCAATTGATGAAACACTTAAGAAGATTATTAGGCATGAAATCA |
| 53 (-) | CCTGTTCCATTTGCCCGAATTTCTTTAGACACATCAGAAAATCTG |
| 54 (-,+) | CCAGGCGTTTCCCCCTGGAAGCTCCCTCGTGCGCTCTCCTGTT  TTTCGCGGGTAATACGCAAAATTAACACCAAGAGGAATTTAACTTAT |
| 55 (+) | CCATTTTCCCTATTGATCCGCAATCGAAAAAGCCCGTGATTTCC  CCAGATGGTAAGCCCTCCCGTATCGTAGTTATCTACACGACGG |
| 56 (+) | CCAGAGCACAAGTTTAATCCCGCCTTTACGCTAAACGGCAAAGG |
| 57 (-,-) | CCGCTGCGCCTTATCCGGTAACTATCGTCTTGAGTCCAACCCGGTAA  CCGCGTGTTATTCAGACTCCCTCTGTATTGCTTTTTCCACGG |
| 58 (-) | CCTAATTTTTCTAACTCTAACGCCCCATAGTTAATCGTGATCCCCA |
| 59 (+) | CCGTCGTTTTACAACGTCGTGACTGGGAAAACCCTGGCGTT  CCCCTTCTTGTCTATCTATGATTACTAGTGCGTCTGAAACTTT |
| 60 (+,-) | AGAATTATCATCTAACGCACGGGCGTGGTTAGAAGGAGAGAAAGA  CCAAAGTATTTGTTGAGTTTGTTAGCAAACGTTTTCAGTGAT |
| 61 (+) | TTTCCCTATTGATCCGCAATCGAAAAAGCCCGTGATTTCCAATTGG |
| 62 (+) | CCATCTCTTTAAGAACTTGATATGAGAGAGGTTTATCCATTGCT |
| 63 (-) | ACATCCGTGACTCCATGGCTTAACAGTACTTTCAACACGTGCCAATC |
| 64 (+) | AACAAATACTTCTTCATACTTGTTATTCCAAAGTATTTGTTGATGG |
| 65 (-) | CCATACACTGTCTATATTATAGAGCCATAGGTCCCTTACTTCTTC |
| 66 (-) | CCTAATTTTTCTAACTCTAACGCCCCATAGTTAATCGTGATCCCCAC |
| 67 (-) | CCTATGGGATATATTAGAATAGAAAAGAAAGGTCATGGAACTAAT |
| 68 (+) | AGAGCAACTCGGTCGCCGCATACACTATTCTCAGAATGACTTGG |
| 69 (+,-) | CCGAAGGGAGAAAGGCGGACAGGTATCCGGTAAGCGGCAGGGTCGG  CCATACACTGTCTATATTATAGAGCCATAGGTCCCTTACTTCT |
| 70 (+) | CCGCCTACATACCTCGCTCTGCTAATCCTGTTACCAGTGGCTGC |
| 71 (-) | CCTTCTCCTAATTTCTTTACCATTCGGATCCTTTCCAAAATACT |
| 72 (-) | CCTTTGATCTTTTCTACGGGGTCTGACGCTCAGTGGAACGAAAACT |
| 73 (-) | CCGCGCCACATAGCAGAACTTTAAAAGTGCTCATCATTGGAAAA |
| 74 (+) | CCTACCCACTGTTTAAAGGCATTAAGTGATTTTGGGCACAATTTT |
| 75 (+) | ATCGGTCCGTTTTTTGCGTAAGCCGTGTTAATATCTGTATAGTAATG  CCATGGTGGTTAAATGGTAAGCACGAAACCAAATGTGCATATTA |
| 76 (+) | CCAGAGCGCAAGTTTAATCCCGCCTATGCGTTAAACGGCAAAG  CCTACACCGAATTGAGATGCCTACAGCGTGAGTTATGAGAAAG |
| 77 (+) | CCTAGAAGAGATATTGAACTATAACAGCCTGGTG |
| 78 (+) | CCAAATACTGTTCTTCTAGTGTAGCCGTAGTTAGGCCACCACTT |
| 79 (-) | AATCTTCTTAACGGTTTCATCAATTGGTTCATAGATTCCCTTT |
| 80 (+) | CCGGGCAAGAGCAACTCGGTCGCCGCATACACTATTCTC |
| 81 (+) | GGTTTATCGAACGAGGCTTTGCCATTTTCCCTATTGATCC |
| 82 (+) | CCATTTTTTAAAACTTTTCCGTTCACTAAAAGCTTTAAATAT |
| 83 (+) | CCGAATGGTAAAGAAATTAGGAGAAGGTGTTTTGTGGGGATC |
| 84 (+) | GTTTTCGTTCCACTGAGCGTCAGACCCCGTAGAAAAGATCAAAGG |
| 85 (+) | TTTTTATGCCATATAACTCATTTTCCATCTCTTTAAAAACTTGATATGAG |
| 86 (-) | TAGAGAGCCTATGGGATATATTAGAATAGAAAAGAAAGGCCA |
| 87 (+) | CCATTGCTAATATTACACTACTTTTCAACATTCTTCACCAAAT |
| Protospacer related to new spacers on locus 2 from genome | |
| 1 (+) | TGCAATCTTCCTTCATAACAGAGAGGAAAGAAGGAAGGTTA |
| 2 (+) | TGAAGGAGAGGGAAGCAGAGGTTATAGGGATGAGTGATGTAGCAAG |
| 3 (+) | CTAGAACCTTAGGCTCATCGACTGGTCCATATATCTTATGACCT |
| 4 (-) | TTGAAGGAAAGGGGAGCAAAGGTTATAGTGATGAGTGATGTAGTAAG |
| 5 (-) | CCAAACAATGTTATCTCTTGACGGAATAGGAGAATGAAAAGCCAATAA |
| 6 (-) | CCACTTGTTTCAGCGACTTCCCAAGGAACGACGGTAATATCAA |
| 7 (+) | CCTTCAATAATTACTGTAATAGGTTATTCAGCAATAGCTGCAGT |
| 8 (-) | CCTTGAGTATCAATCATCTATTGTGAGCCAAGAGAAAAAAGTTAG |
| 9 (+) | CCAGAATGGAGGGCATTTACTCCAGAAGAGAAAGAAAAGA |

(+): protospacer on coding strand, (-): protospacer on non-coding strand; PAM sequences are underline, the adjacent sequences of protospacers with no PAM are in blue letters.
